# Portacaval Shunt Established in Six Dogs Using Magnetic Compression Technique

**DOI:** 10.1371/journal.pone.0076873

**Published:** 2013-09-30

**Authors:** Xiaopeng Yan, Chao Fan, Jia Ma, Jianhui Li, Dinghui Dong, Haohua Wang, Feng Ma, Xinglong Zheng, Yi Lv

**Affiliations:** 1 Department of Hepatobiliary Surgery, First Affiliated Hospital, College of Medicine, Xi’an Jiaotong University, Xi’an Shaanxi Province, China; 2 Department of General Surgery, Tangdu Hospital, Fourth Military Medical University, Xi’an Shaanxi Province, China; 3 Department of Surgical Oncology, Third Affiliated Hospital, College of Medicine, Xi’an Jiaotong University (Shaanxi Provincial People’s Hospital), Xi’an Shaanxi Province, China; 4 XJTU Research Institute of Advanced Surgical Technology and Engineering, Xi’an Jiaotong University, Xi’an Shaanxi Province, China; UNIFESP Federal University of São Paulo, Brazil

## Abstract

**Background and Aims:**

Installing the transjugular intrahepatic portosystemic shunt for portal hypertension is relatively safe, but complications are still high. To explore a new method of portacaval shunt, the magnetic compression technique was introduced into the shunting procedure.

**Methods:**

A portal-inferior vena cava shunt was performed on 6 male mongrel dogs by two hemocompatible Nd-Fe-B permanent magnets, parent and daughter. The parent magnet was applied to the inferior vena cava guided by a catheter through the femoral vein. The daughter magnet was moved to the anastomosis position on the portal vein with a balloon catheter through the splenic vein. After the daughter magnet reached the target position, the two magnets acted to compress the vessel wall and hold it in place. Five to 7 days later, under X-ray guidance, the magnets were detached from the vessel wall with a rosch-uchida transjugular liver access set. One month later, histological analysis and portal venography were performed.

**Results:**

5-7 days after the first surgery, a mild intimal hyperplasia in the portal vein and the inferior vena cava, and continuity of the vascular adventitia from the portal vein to the inferior vena cava as observed. During the second surgery, the contrast media could be observed flowing from the portal vein into the inferior vena cava. Portal venography revealed that the portosystemic shunt was still present one month after the second surgery.

**Conclusions:**

Magnamosis via a device of novel design was successfully used to establish a portacaval shunt in dogs.

## Introduction

Portal hypertension, the most common complication of cirrhosis, is defined as portal vein pressures exceeding 5 mm Hg or portal vein to hepatic vein gradient of greater than 10 mm Hg. The most serious complication of portal hypertension is variceal bleeding with a gradient above 12 mm Hg, usually in the esophagus or stomach [1]. The risk of bleeding in patients with large varices is approximately 30% over 2 years [2]. Decompression of varices can be achieved with surgical shunts including portacaval shunt, and a number of randomized studies comparing different surgical shunts in patients have been published. Currently, transjugular intrahepatic portosystemic shunt (TIPS) is considered a standard treatment for complications related to portal hypertension, including variceal bleeding [3]. All types of surgical shunts prevented rebleeding in >90% of patients. However, these surgeries share several shortcomings, for example, complicated microsurgery, restenosis of the shunt etc.

Magnetic compression is a minimally invasive treatment that, in conjunction with an interventional radiologic technique, uses two high-power magnets to create a nonsurgical, sutureless enteric anastomosis. Surgical innovators have pursued the possibility of compression anastomosis ever since it was first conceived by Denan in 1826 [4,5]. In 1978, Obora et al. [6] developed a method for non-suture microvascular anastomosis using a magnetic compression device, in which an average of 8.3 minutes was required to create an anastomosis with a high rate of patency, and histologically, a continuity of vascular wall was noted.

In recent years the magnetic compression technique (MCT) has been applied to solve many surgical problems, including malignant bowel obstruction, enterostomy closure, traumatic bowel injury, and congenital anomalies of the esophagus and intestine [7]. Previously we proposed a simple, timesaving, safe, and efficient method for performing Roux-en-Y choledochojejunostomy procedures using magnetic compression in a canine model of obstructive jaundice [8]. We also confirmed that magnetic rings could be used for rapid vascular reconstruction in a canine liver transplantation model [9].

This study aims to create the vascular shunt without stent and the inner wall of the shunt will be covered by endothelium. Then, the restenosis may be some how avoid as there was no foreign bodies had been planted in. As MCT was successfully used in many organs, then, we hypothesized that MCT can be used to set up a vascular anastomosis between portal vein and inferior vena cava when necessary. In the present study we described magnamosis (magnetic compression anastomosis) with a novel device for establishing a portacaval shunt in dogs.

## Materials and Methods

### Magnetic Compression Device

**Figure 1:**
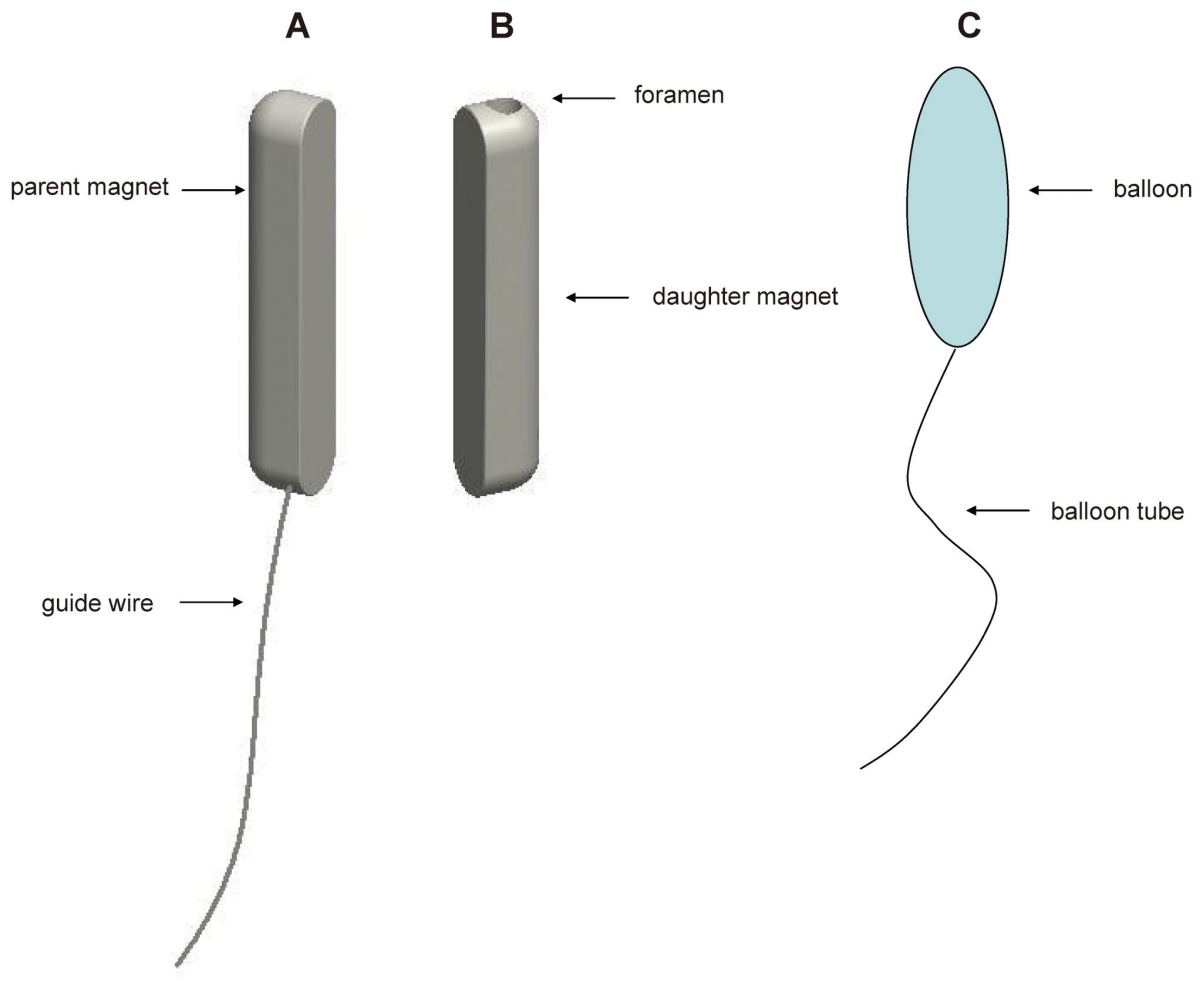
Schematic diagram of the magnetic compression system. The magnetic compression system for portacaval shunt includes **A**, a parent magnet attached to an anchor wire and **B**, a daughter magnet. **C**, The balloon catheter can be inserted into the middle hole of the daughter magnet when the balloon as not inflated with saline. After the balloon is inflated, the daughter magnet can be fixed to the balloon catheter.

Two hemocompatible Nd-Fe-B permanent magnets (parent and daughter) with a polyurethane/heparin/paclitaxel coating were specifically manufactured for experimental vascular use (Northwest Institute for Nonferrous Metal Research, Xi’an, China; Figure 1). The strength of the magnet is 5.4N with magnetic density of 2600GS. The weight of parent and daughter magnet is 0.32 g and 0.30 g, respectively. The brief protocol for the coating is as follows, 1: polyurethane (2 g) was added into 100ml tetrahydrofuran mix and shaking 24 hours. 2: Heparin (100 mg) and carbodiimide (20 mg) were added and vortex for 10min. 3: the magnets were dipped in the solution for 3 minutes. 4: The dipped magnets were vacuum dried for 24 hours. The parent magnet is a semi-cylinder with round ends, and length, width, and thickness are 12, 2.5, and 2.5 mm, respectively. The parent magnet is attached to a special anchor wire, which was modified from a 2-0 prolene wire (Ethicon, Johnson & Johnson, Somerville, NJ, USA) by cutting away the suture needles from its ends. In order to put the parent magnet into the target position, the left femoral vein was isolated and the ligated at the distal end. After that, venotomy was performed and the parent magnet is introduced from the femoral vein into the inferior vena cava by the anchor wire.

The daughter magnet, without wire, has a hole (diameter, 1.2 mm) through the center that is parallel to the long axis. Like the parent magnet, it is a semi-cylinder with round ends and length, width, and thickness are 12, 2.5, and 2.5 mm, respectively. The balloon catheter is attached to the daughter magnet by inserting the balloon into and through the middle hole of the daughter magnet. The daughter magnet is introduced into the portal vein through the isolated splenic vein with the ligated distal end by using a catheter with a balloon (2.5 mm x 15 mm long) (Medtronic, USA) at the end.

### Surgical Procedure

This study was carried out in strict accordance with the recommendations in the Guide for the Care and Use of Laboratory Animals of Xi’an Jiaotong University Medical Center. The protocol was approved by the Committee on the Ethics of Animal Experiments of Xi’an Jiaotong University (Permit Number: 2010-105). All surgery was performed under sodium pentobarbital anesthesia, and all efforts were made to minimize suffering. Six male mongrel dogs >1-year-old and weighing 12-15 kg were obtained from the Experimental Animal Center, College of Medicine, Xi’an Jiaotong University (Xi’an, China).

Each animal was intraperitoneally anesthetized with pentobarbital (30 mg/kg), after fasting for 12 h and abstinence from water for 6 h. With the animal in a supine position, the upper abdomen, right lateral crural region, medial thigh, and groin were shaved and draped with sterile towels. Lactate Ringer’s was administrated (500 mL intravenous) through the right great saphenous vein. The common portal vein and inferior vena cava were exposed through an abdominal incision. The splenic vein and 15 mm of the left femoral vein were isolated. The femoral vein was occluded at the distal end. The prolene wire affixed to the parent magnet was inserted into a 5F Cook catheter (Figure S1, Cook, Bloomington, IN, USA). The end of the prolene wire was tightly drawn to make sure the parent magnet was fixed to the other end of the Cook catheter. Five minutes before inserting the magnets, a heparin sodium injection was administrated (1 mg/kg) through the right great saphenous vein to prevent thrombosis. A phlebotomy was performed at the proximal part of the femoral vein. The parent magnet was then applied to the target position for vessel anastomosis on the inferior vena cava, guided by a Cook catheter through the incision on the femoral vein.

**Figure 2:**
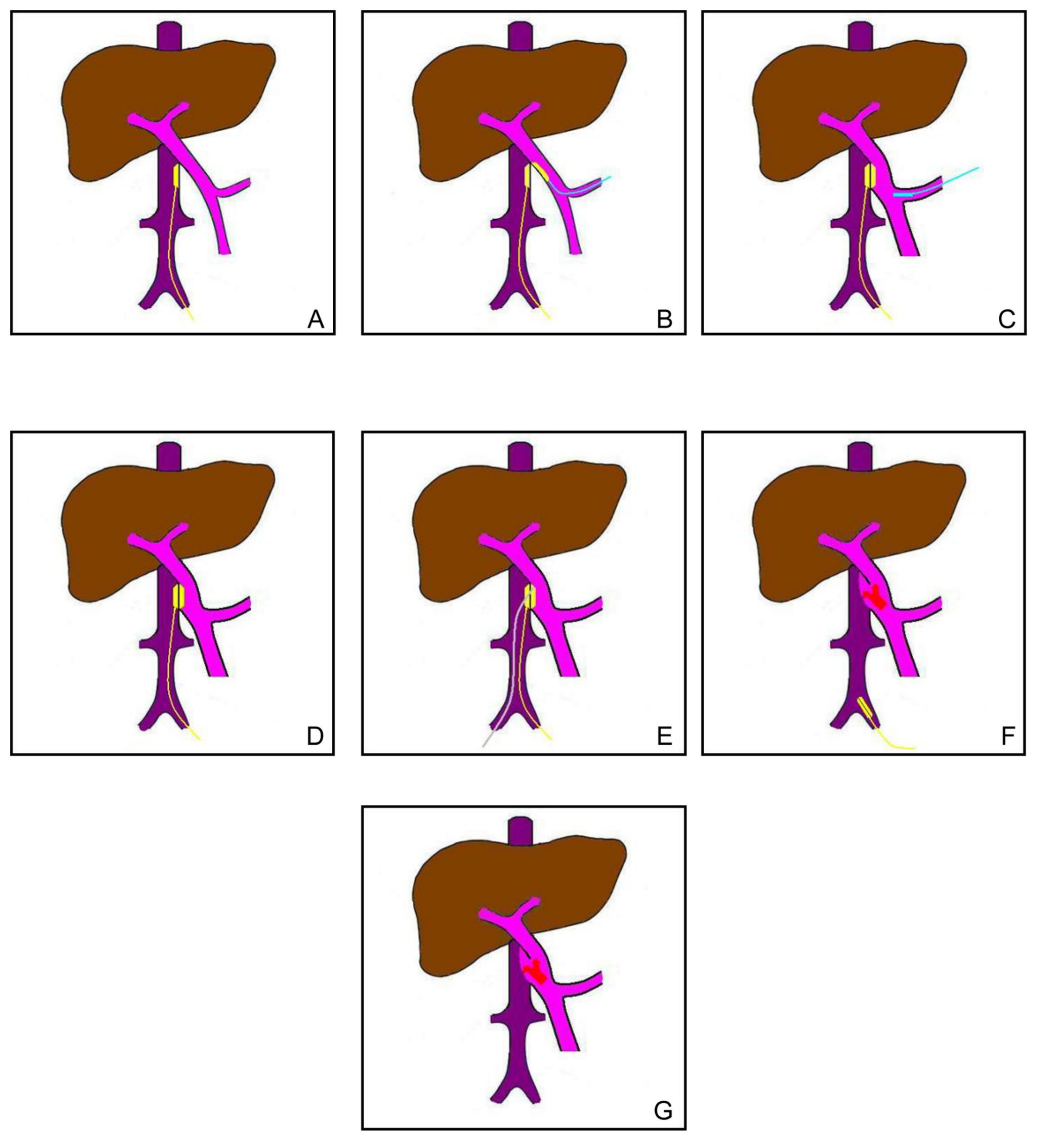
Surgical protocol. **A**, The parent magnet was guided to the target position for vessel anastomosis on the inferior vena cava by the wire fixed to one end. **B**, The daughter magnet was moved to the anastomosis position on the portal vein by the balloon catheter through the incision on the splenic vein. **C**, After the parent magnet met the daughter magnet, the balloon catheter was withdrawn from the vessel. **D**, The position of the magnets and the wire after the first surgery. **E**, Five to 7 days after the first surgery, a rosch-uchida transjugular liver access set inserted in a vascular sheath was introduced into the position of magnet in the inferior vena cava through the right femoral vein. Under X-ray guidance, the needle of the rosch-uchida transjugular liver access set was advanced slowly and ceaselessly along the outline of the magnets and the magnets were detached from the vascular wall. **F**, The magnets were pulled out of the body by the wire which was fixed to the parent magnet. **G**, The portal-inferior vena cava shunt was set up.

**Figure 3:**
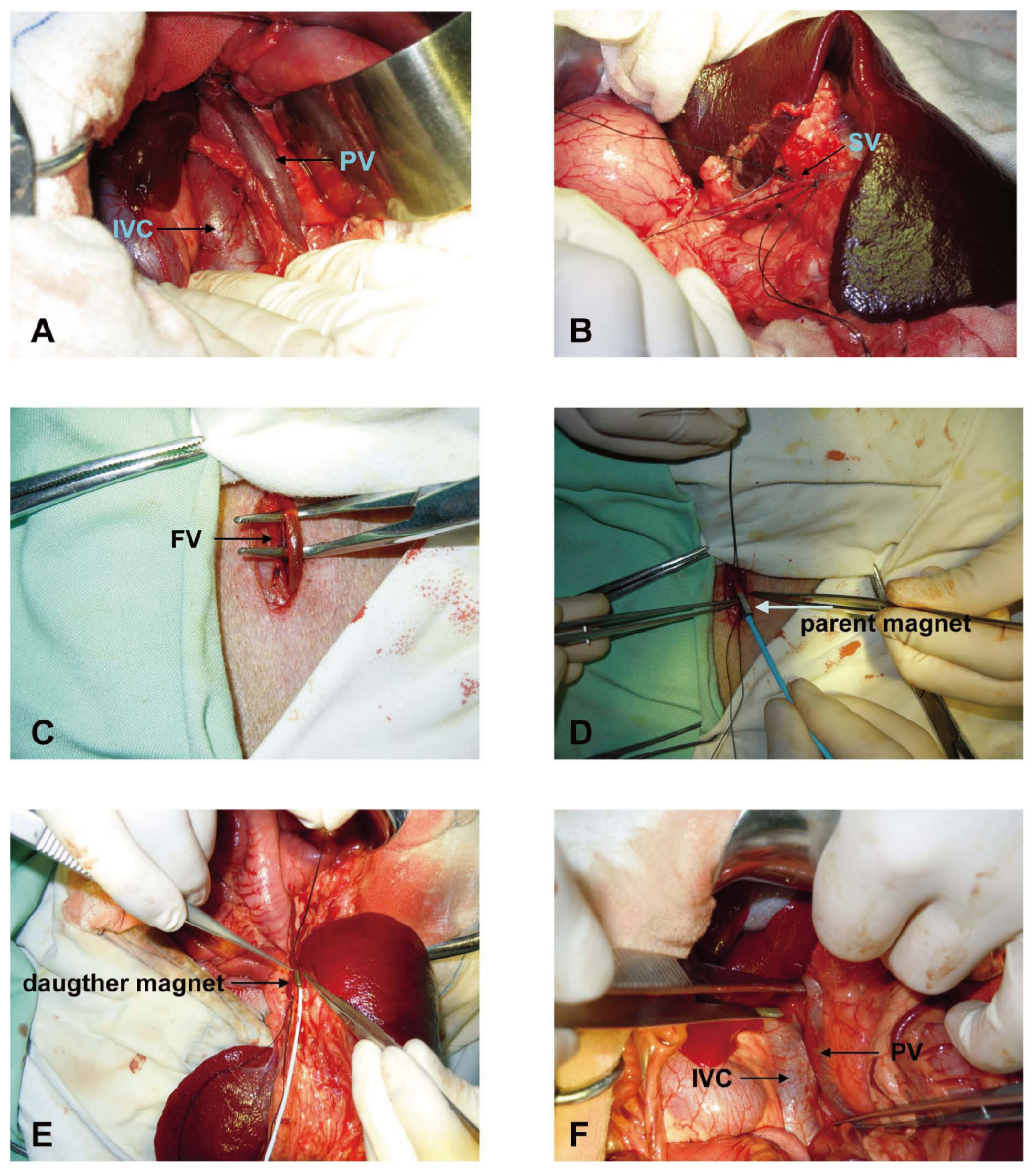
Procedure of the magnamosis surgery. **A**, Inferior vena cava and portal vein. **B**, Isolated splenic vein. **C**, Isolated femoral vein. **D**, The parent magnet was inserted into the femoral vein with incision. **E**, The daughter magnet was pulled into the splenic vein. **F**, The parent magnet clung to the daughter magnet and compressed the anastomosis site.

A balloon catheter was inserted through the hole of the daughter magnet. The daughter magnet was fixed to the balloon catheter by filling the balloon with saline. A venotomy was performed on the 15 mm-long isolated splenic vein, which was occluded at the distal end. The daughter magnet was moved to the anastomosis position on the portal vein using the balloon catheter through the incision on the splenic vein. After the daughter magnet reached the target position, the attraction between the two magnets compressed the vessel wall between them to hold it in place. The balloon catheter was then retreated from the daughter magnet by releasing the saline from the balloon and pulling it out of the splenic vein, which resulted in the detachment of the magnet from the balloon catheter. The Cook catheter was withdrawn from the wire fixed to the parent magnet and pulled out of the left femoral vein. The incision in the femoral vein was ligated at the proximal side and the wire connected to the parent magnet was carefully fixed to the ligation (Figure 2A-C and Figure 3). Finally, the wounds were closed with suture for the muscle, subcutaneous layers, and the cutaneous layer. Postoperatively the dogs received subcutaneous 5000 U/day dalteparin (Vetter Pharma-Fertigung GmbH & Co KG, Germany) until the third day after the second surgery, plus intramuscular penicillin 1000 mg twice daily for 3 continuous days.

**Figure 4:**
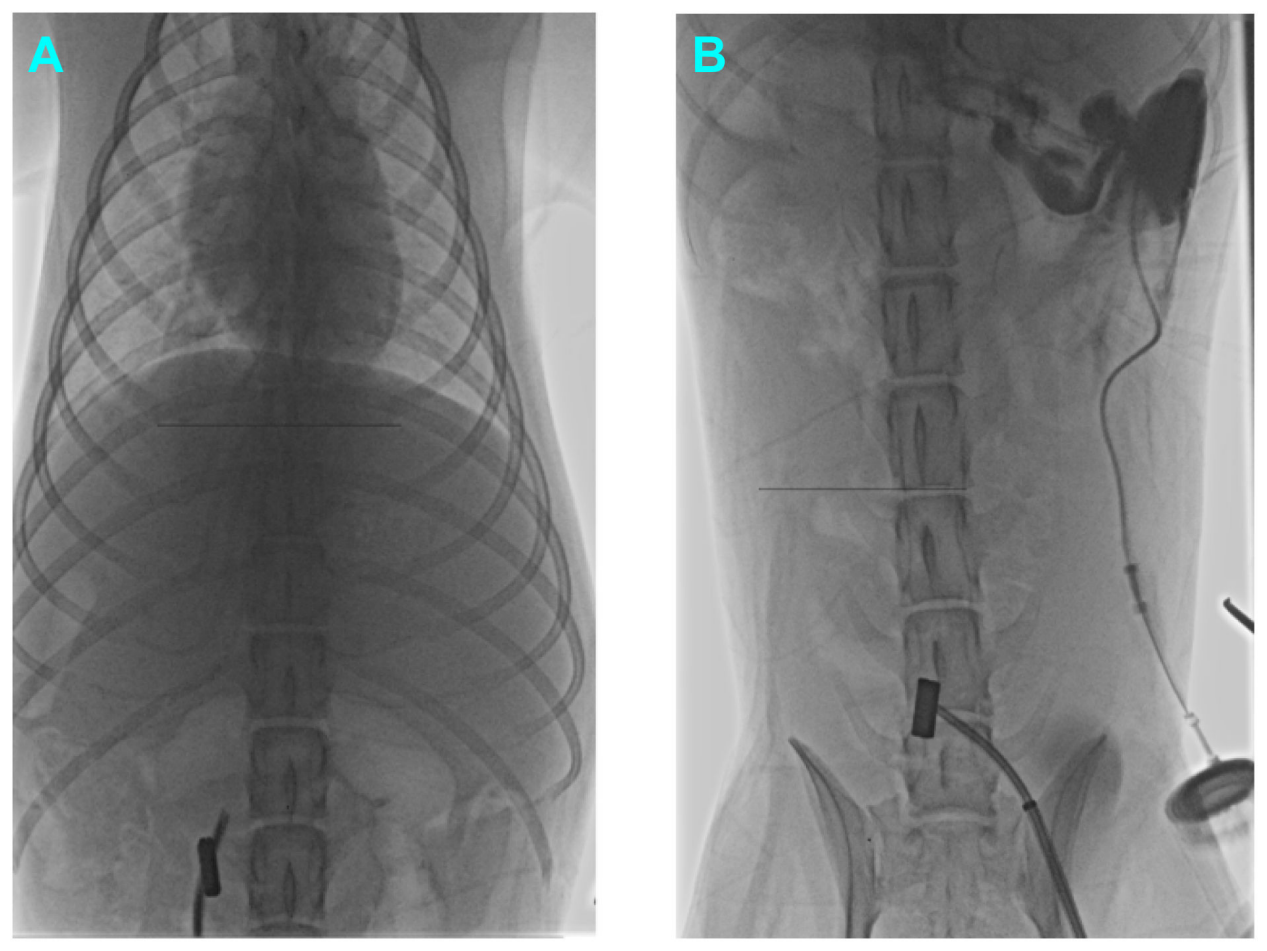
Procedure of the second surgery. **A**, Under X-ray guidance, the needle of the rosch-uchida transjugular liver access set was advanced slowly and ceaselessly along the outline of the magnets. **B**, The magnets were pulled out of the body by the wire fixed to the parent magnet (X-ray photograph).

**Figure 5:**
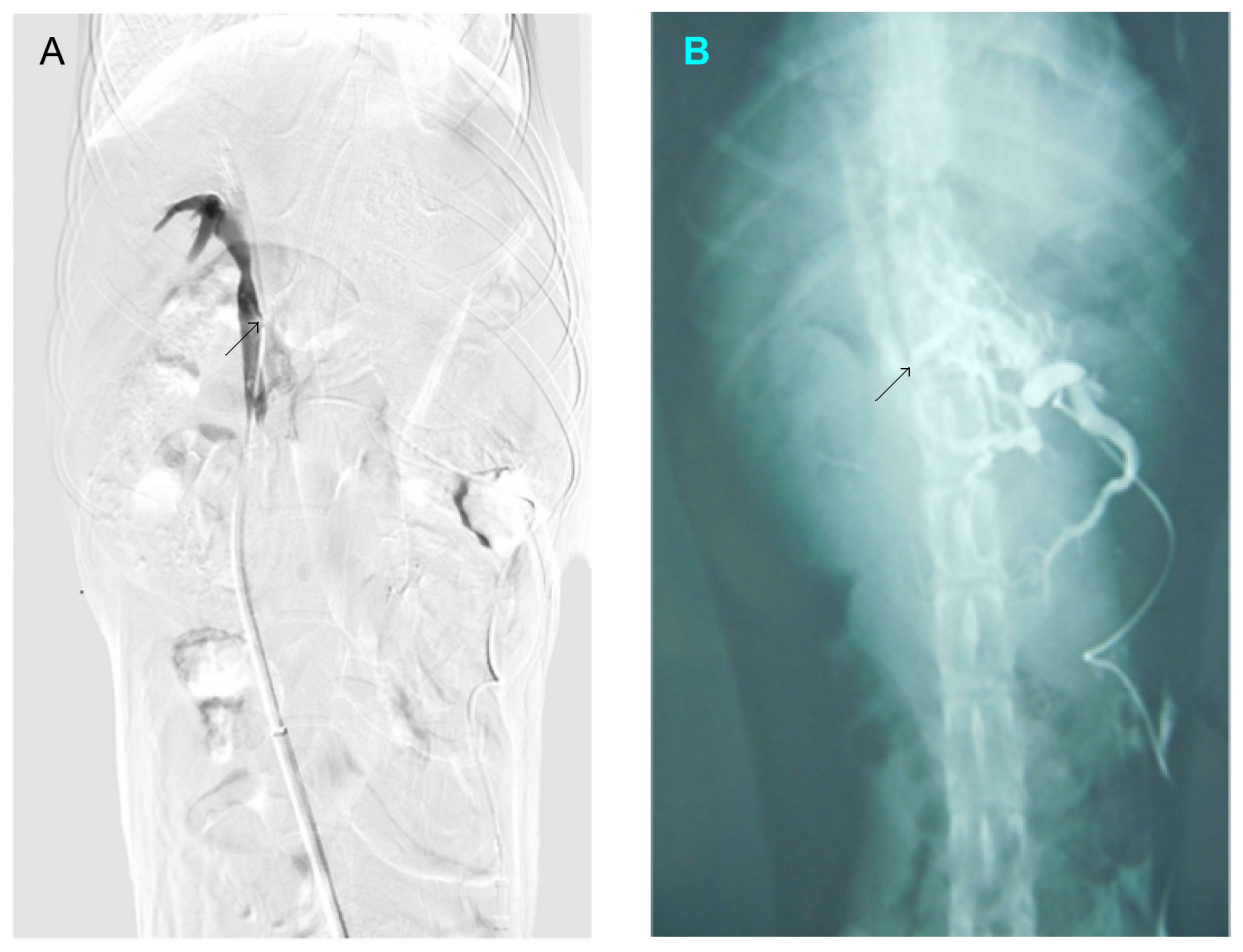
Portography during the second surgery and after the surgery. **A**, During the second surgery, the contrast media can be observed flowing from the portal vein into the inferior vena cava. **B**, Portal venography revealed that the portosystemic shunt still existed one month after the second surgery.

Based on the data from the preparation experiments, five to 7 days after the first surgery the second surgery was performed. After cutdown and dilation of the right femoral vein, the 10F sheath of a rosch-uchida transjugular liver access set (Cook, Bloomington, IN, USA) was introduced into the position of the magnet in the inferior vena cava. Under X-ray guidance, the trocar needle of the rosch-uchida transjugular liver access set was punctured into the left femoral vein and enters the vagina vasorum. After the needle reached the position of parent magnet in the inferior vena cava, the direction of the tip of the needle was adjusted to the portal vein. Then, the tip of the needle advanced slowly but without stopping along the outline of the magnets. When the magnets detached from the vessel wall, the magnets together with the anchor wire fixed to the parent magnet at the first surgery was pulled back slowly from the left femoral vein (Figure 2D-G and Figure 4). Then, portal venography was performed immediately and a successful shunt of the portal vein-inferior vena cava could be observed (Figure 5A). After that, the rosch-uchida transjugular liver access set and the sheath was removed and the femoral veins were ligated.

### Histological Analysis

One month after the second surgery, the dogs were anesthetized with pentobarbital and portography was performed. The portosystemic shunt position was carefully exposed and collected after the portography and anesthetization of the animals. Five millimeters from the portosystemic shunt position, the portal vein and the inferior vena cava were cut and the portosystemic shunt vessels were immersed in 10% buffered formalin overnight. After fixation, vessels were then embedded in paraffin, and 4-µm thick sections were cut at the site of the anastomosis. Sections were stained with H&E or Masson’s trichrome stain and examined under a bright-field microscope. 

## Results

**Figure 6:**
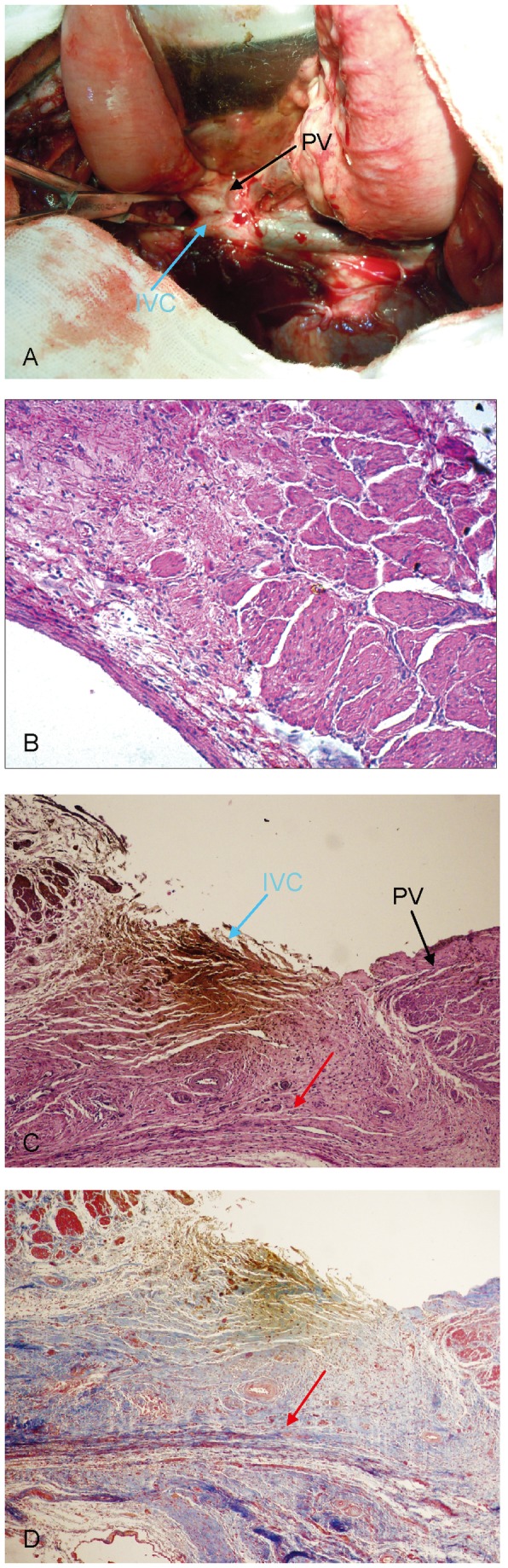
Histology of the portal-inferior vena cava shunt. **A**, The two magnets stably matched and a large number of fibrous connective tissue can be observed around the anastomosis. **B**, Mild intimal hyperplasia in the portal vein and the inferior vena cava (H&E staining, 40×). **C**, Continuity of the vascular adventitia from the portal vein to the inferior vena cava (H&E staining, 40×). **D**, A large quantity of tidily arranged collagen surrounding the adventitia (Masson’s trichrome stain, 40×). Red arrow: continuous fusion between the portal vein and the vascular adventitia of the inferior vena cava.

The surgery was successfully performed on all six dogs, and all survived the entire experimental period without any observable complications, bleeding, or thrombosis. During the first procedure, after the two magnets were placed into the target positions they pressed together immediately. We found that 5 to 7 days after the first surgery was the best time to perform the second surgery; at this time, a large amount of fibrous connective tissue had formed and could be observed around the anastomose. H&E staining of the anastomose revealed a mild intimal hyperplasia in all the dogs in the portal vein and the inferior vena cava, a continuity of the vascular adventitia from the portal vein to the inferior vena cava. Masson’s trichrome stain revealed a large quantity of tidily arranged collagen surrounding the adventitia (Figure 6).

During the second surgery, the contrast media could be observed flowing from the portal vein into the inferior vena cava and the length of the shunt is around 12 mm and the width of the shunt is approximate 2.5 mm. One month after the second surgery, portal venography revealed that the portosystemic shunt was still present (Figure 5B). The length of the shunt is around 10 mm and the width of the shunt is approximate 3 mm.

## Discussion

To better observe the progress of the surgery, in the present study we opened the abdomen. However, our method does not require this and minimally invasive surgery can be successfully achieved. In the past, a number of surgical protocols that required opening the abdomen were developed to manage portal hypertension. These can be classified in general as either shunting or non-shunting [10]. The goal of the former is to reduce the incidence of variceal bleeding by lowering the pressure in the portal system via the creation of a portosystemic shunt [11]. The latter were developed to decrease the high rates of encephalopathy associated with portosystemic anastomoses. However, non-shunting procedures always include a splenectomy, and because the spleen is a crucial immunological organ, shunts, especially TIPS, are more widely used to solve the problem of portal hypertension [12].

Many patients with portal hypertension have low liver function, hypocoagulability, and low levels of serine and cannot tolerate surgery and anesthesia; they are therefore not able to benefit from shunting surgery [13]. To achieve a minimally invasive surgery with long-term shunting effects, we introduced magnetic compression into the shunting procedure for the treatment of portal hypertension. The unique aspects of the magnamosis (magnetic anastomosis) device include a topology specific for the purpose and application of a calibrated magnetic field [14,15]. To form an anastomosis by transluminal compression, the two magnets must be suitably designed so that the tissue can remodel and form a ring around the new anastomotic channel [16]. Our work demonstrates the feasibility of performing sutureless side-to-side compression anastomoses using well-designed magnetic attraction. Neither leaks nor appreciable stenoses were observed during the observation period.

With the development of covered stents, the frequency of TIPS dysfunctions has been reduced [17]. However, the incidence of hyperplastic stenosis is still one of TIPS’ main shortcomings [18]. Because the stent is not used in the protocol we describe here, hyperplastic stenosis should not occur. Furthermore, the absence of fiber or wire foreign bodies allows the anastomoses to expand dynamically in response to fluctuations of pressure, potentially decreasing the likelihood of anastomotic stricture or obstruction.

In our experiments, the timing of the second surgery is crucial for a successful result. If there is not enough time for remolding, bleeding will be inevitable during the second surgery. However, if the second surgery is performed too long after the first, the magnets will become embedded in adhesive tissues and will be difficult to remove. Based on our experience, 5 to 7 days after the first surgery is the working window for the second surgery in dogs. Additionally, we also detected the portal pressure, blood ammonia and liver function before and after the portacaval shunt was established (Figure S2). Although the dog with normal portal pressure was used, we still can observe the significantly reduced portal pressure after the portacaval shunt was established (p<0.01). At the same time, the blood ammonia and liver function was not significant changed before and after the portacaval shunt was established indicating the diameter of the shunt established by our technique is suitable and did not lead to liver dysfunction and hepatic encephalopathy. Our results suggest that magnamosis is feasible.

Hepatic encephalopathy and TIPS dysfunction are the two most significant complications that have limited the effectiveness of TIPS [19]. TIPS diverts portal blood flow from the liver resulting increased hepatofugal flow and increases the risk of encephalopathy [20]. In some cases, the calibre of the shunt has to be reduced to limit hepatofugal flow when the encephalopathy cannot respond to standard therapy. Although covered stents reduced the rate of shunt dysfunction, the potential for development of endothelium vascular hyperplasia is still complications that can cause TIPS dysfunction [21]. Based on the data presented in this study, the size of the shunt may be controlled by the size of the magnets. Furthermore, our preliminary data showed that the inner wall of the vascular shunt was covered by endothelium after the daughter-parent magnets were pulled out implying the possibility of less vascular hyperplasia of our method. However, we only presented a new method (idea) in this study and the data is preliminary. Future work is needed to confirm and improve the efficiency of this method in minimally invasive surgery with longer observation time.

There are several limitations that need to be acknowledged and addressed regarding the present study. 1) requirement for two surgical procedures and two separate hospitalizations/anesthesia exposure for potential patients; 2) increasing risk for peritoneal infection via cut-downs required - particularly splenic access; 3) difficulty in monitoring the status of the shunt using ultrasound due to overlying bowel/gut; 4) Because of the short observation period, it is till hard to safetly say this method reduces the risk of hyperplastic stenosis; 5) This is an initial model to assess safety of venous anastomosis in normal pressure regimens in non-cirrhotic livers, future experiment in cirrhotic livers was needed. However, this preliminary study confirmed part of idea, that is, a vascular anastomosis between portal vein and inferior vena cava can be set up without stent use. At least, we can safely say that 1) this method do not leave foreign bodies in the animals and do not need longtime usage of anticoagulants; 2) laparotomy can be avoided by intervention technology (percutaneous portal vein puncture) in the future based on our method.

In conclusion, we used a unique magnamosis device and magnetic compression to establish a portacaval shunt in dogs. This may be useful as surgical therapy for portal hypertension, even for vascular surgery and reconstructive surgery.

## Supporting Information

Figure S1 **The magnetic compression device.** A: 5F Cook catheter, parent magnet and the prolene wire; B: the 5F Cool catheter; C: the parent magnet, the small scale: 1 mm; D: parent magnet with the prolene wire inserted into the 5F Cook catheter.
(TIF)

Figure S2 **Portal vein pressure, liver function and blood ammonia test.** A: Portal vein pressure before and 30 days after the Portacaval Shunt was established. B: blood levels of alanine aminotransferase (ALT), aspartate transaminasec (AST), total protein (TP) and Albumin (ALB) before and 7 or 30 days after the Portacaval Shunt was established. C: Blood ammonia concentration before and 7 or 30 days after the Portacaval Shunt was established. *, p<0.01, as compared to the group before the Portacaval Shunt was established, n=6.
(TIF)
