# Supplementary figures and images for: Portacaval Shunt Established in Six Dogs Using Magnetic Compression Technique
Source: PLoS One. 2013 Sep 30;8(9):e76873. doi: 10.1371/journal.pone.0076873 (PMC3786958; doi:10.1371/journal.pone.0076873)

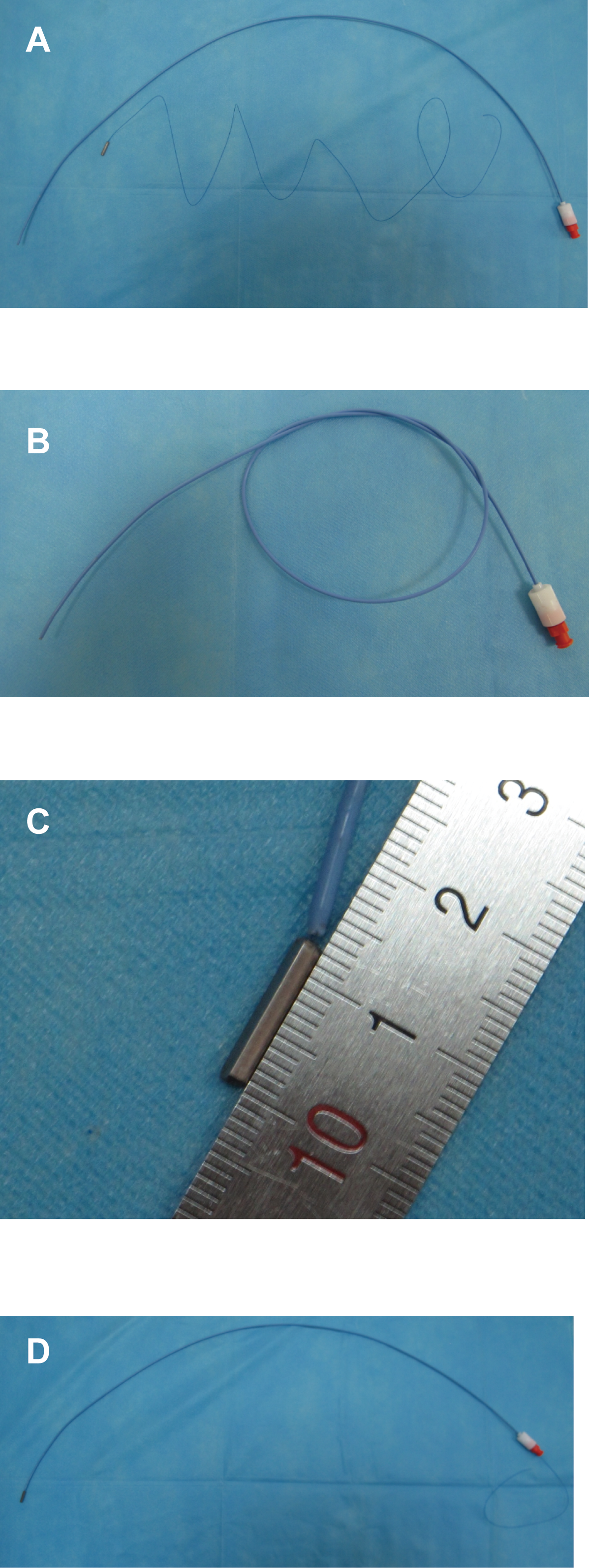

Supplement: Figure S1 — The magnetic compression device. A: 5F Cook catheter, parent magnet and the prolene wire; B: the 5F Cool catheter; C: the parent magnet, the small scale: 1 mm; D: parent magnet with the prolene wire inserted into the 5F Cook catheter. (TIF) [file pone.0076873.s001.tif]

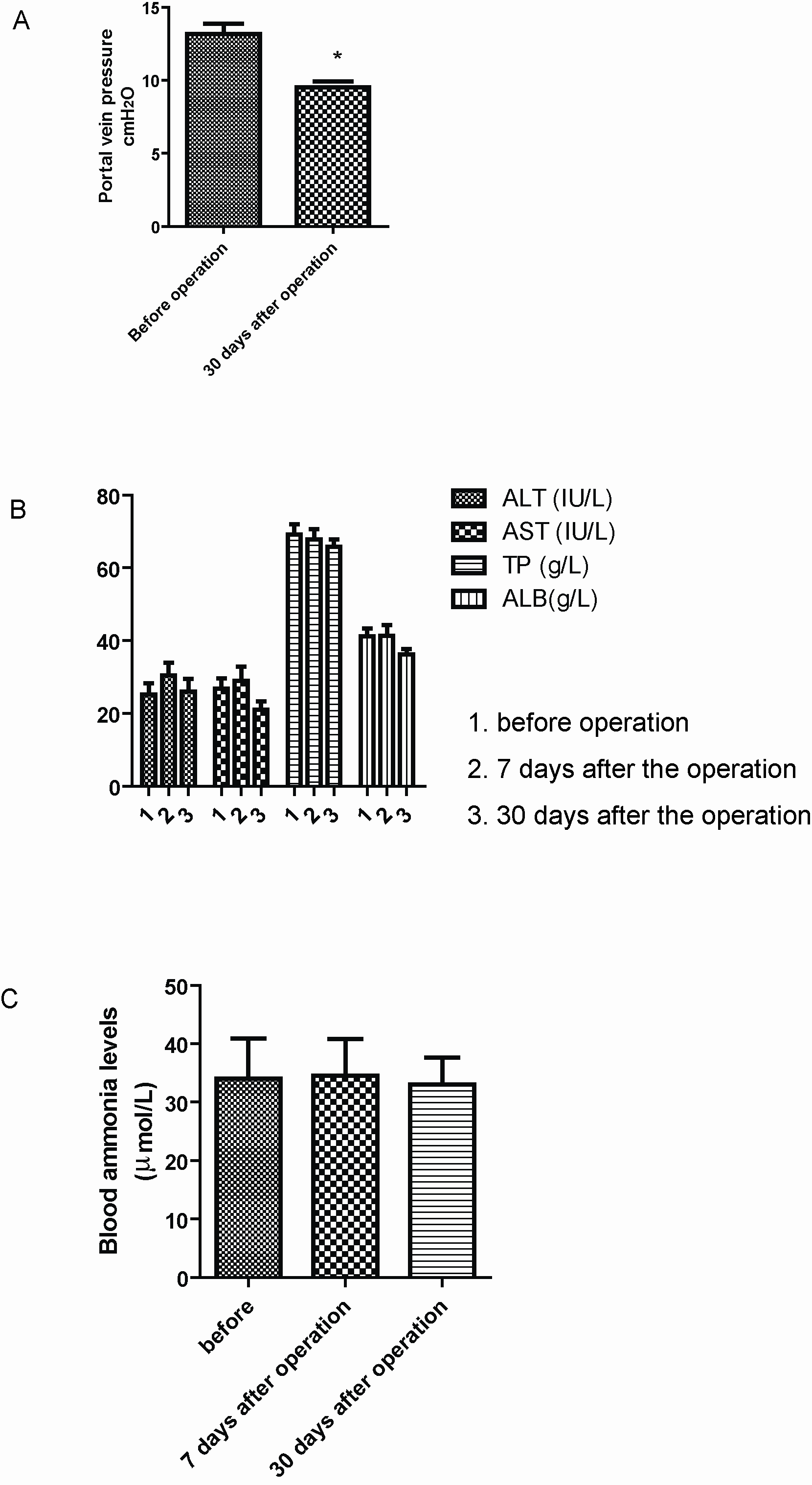

Supplement: Figure S2 — Portal vein pressure, liver function and blood ammonia test. A: Portal vein pressure before and 30 days after the Portacaval Shunt was established. B: blood levels of alanine aminotransferase (ALT), aspartate transaminasec (AST), total protein (TP) and Albumin (ALB) before and 7 or 30 days after the Portacaval Shunt was established. C: Blood ammonia concentration before and 7 or 30 days after the Portacaval Shunt was established. *, p<0.01, as compared to the group before the Portacaval Shunt was established, n=6. (TIF) [file pone.0076873.s002.tif]
